# Resistance to the Novel Fungicide Pyrimorph in *Phytophthora capsici*: Risk Assessment and Detection of Point Mutations in CesA3 That Confer Resistance

**DOI:** 10.1371/journal.pone.0056513

**Published:** 2013-02-19

**Authors:** Zhili Pang, Jingpeng Shao, Lei Chen, Xiaohong Lu, Jian Hu, Zhaohai Qin, Xili Liu

**Affiliations:** 1 Department of Plant Pathology, College of Agriculture and Biotechnology, China Agricultural University, Beijing, China; 2 Department of Applied Chemistry, College of Science, China Agricultural University, Beijing, China; Seoul National University, Republic of Korea

## Abstract

Pyrimorph is a novel fungicide with high activity against the plant pathogen *Phytophthora capsici.* We investigated the risk that *P. capsici* can develop resistance to pyrimorph. The baseline sensitivities of 226 *P. capsici* isolates, tested by mycelial growth inhibition, showed a unimodal distribution with a mean EC_50_ value of 1.4261 (±0.4002) µg/ml. Twelve pyrimorph-resistant mutants were obtained by repeated exposure to pyrimorph *in vitro* with a frequency of approximately 1×10^−4^. The resistance factors of the mutants ranged from 10.67 to 56.02. Pyrimorph resistance of the mutants was stable after 10 transfers on pyrimorph-free medium. Fitness in sporulation, cystospore germination, and pathogenicity in the pyrimorph-resistant mutants was similar to or less than that in the parental wild-type isolates. On detached pepper leaves and pepper plants treated with the recommended maximum dose of pyrimorph, however, virulence was greater for mutants with a high level of pyrimorph resistance than for the wild type. The results suggest that the risk of *P. capsici* developing resistance to pyrimorph is low to moderate. Among mutants with a high level of pyrimorph resistance, EC_50_ values for pyrimorph and CAA fungicides flumorph, dimethomorph, and mandipropamid were positively correlated. This indicated that point mutations in cellulose synthase 3 (CesA3) may confer resistance to pyrimorph. Comparison of CesA3 in isolates with a high level of pyrimorph resistance and parental isolates showed that an amino acid change from glutamine to lysine at position 1077 resulted in stable, high resistance in the mutants. Based on the point mutations, an allele-specific PCR method was developed to detect pyrimorph resistance in *P. capsici* populations.

## Introduction

*Phytophthora capsici* Leonian is a heterothallic oomycete pathogen that was first described by Leon H. Leonian in 1922 [1]. It affects all cucurbits, a wide range of solanaceous plants, and snap (*Phaseolus vulgaris* L.*)* and lima beans (*Phaseolus limensis* Macf.) worldwide, causing crown, root, and fruit rot [2]–[13]. When weather favors *P. capsici*, devastating crop losses may occur [1], [12], [13]. Fungicides are recommended to control *P. capsici*-induced diseases, in combination with other management practices, such as crop rotation, raised beds, and water management [12]. Although phenylamide fungicides (e.g., metalaxyl) have been widely used for *P. capsici* control for many years in China, fungicide resistance is a major challenge. When the pathogen is resistant to metalaxyl or mefenoxam [14]–[19], a limited number of fungicides are available for combating *P. capsici*.

Pyrimorph, (*E*)-3-[(2-chloropyridine-4-yl)-3-(4-*tert*-butylphenyl) acryloyl] morpholine, is a novel fungicide that was synthesized in China in 2003 [20]. It exhibits excellent activity against *Rhizoctonia solani* and some oomycetes such as *Phytophthora infestans*, *Pseudoperonospora cubensis, Peronophythora litchii*, and *P. capsici* [21]–[24]. In sensitive oomycetes, pyrimorph strongly inhibits various asexual stages including mycelial growth, sporangium production, and cystospore germination [23], [24]. Pyrimorph is currently registered in China for the control of *Phytophthora* blight of pepper (*Capsicum annuum* L.) and tomato (*Lycopersicum esculentum* Mill) late blight (http://www.chinapesticide.gov.cn). About 20,000 kg of the active ingredient in pyrimorph is produced each year by the Jiangsu Gengyun Company in China, and the recommended application dose in the field ranges from 375 to 450 g ha^−1^. Pyrimorph production and application will increase in the near future [25].

Pyrimorph’s mechanism of action is currently the subject of intensive investigation. Inhibition of *P. capsici* by pyrimorph might result from multiple modes of action including the impairment of the energy generation system and direct or indirect impairment of cell wall biosynthesis [23]. The mechanism of action for pyrimoph might be similar to that for carboxylic acid amide (CAA) fungicides because CAA-resistant laboratory mutants of *P. capsici* were also resistant to pyrimorph [26]. Recent research has identified amino acid changes in cellulose synthase 3 (CesA3) that confer resistance to CAA fungicides in the following oomycetes: *Plasmopara viticola*, *Phytophthora infestans*, *Pseudoperonospora cubensis*, and *Phytophthora melonis* [27]–[31]. However, the resistance mechanism of *P. capsici* to pyrimorph has not been elucidated at the molecular level.

Although pyrimorph has been marketed in China for about 3 years, it is still in the early stages of use on pepper, and resistance of *P. capsici* to pyrimorph has not yet been reported. Determining the response profiles of *P. capsici* field populations to pyrimorph and studying the characteristics of laboratory-generated mutants with pyrimorph resistance will be useful for estimating the risk that *P. capsici* can develop resistance to this new fungicide [32]. In the current study, we established the baseline sensitivity of *P. capsici* from 40 locations in 24 provinces of China to pyrimorph; we generated pyrimorph-resistant mutants with stable resistance by exposing isolates to pyrimorph *in vitro*; and we then assessed the risk that *P. capsici* can develop resistance to pyrimorph. By assaying cross-resistance between pyrimorph and other oomycete fungicides, we speculated that pyrimorph has a similar resistance mechanism to CAA fungicides, and we identified a novel point mutation at codon position 1077 in the *PcCesA3* gene that is linked to pyrimorph resistance. Based on this mutation, a rapid and reliable allele-specific PCR method was developed to detect pyrimorph-resistant isolates in populations of *P. capsici*.

## Materials and Methods

### Fungicides and Media

Technical-grade fungicides included pyrimorph (95% active ingredient [a.i.], kindly provided by Professor Qin, China Agricultural University, China), dimethomorph (95% a.i., Frey Agrochemicals Ltd., Jiangsu, China), flumorph (92.5% a.i., Research Institute of Chemical Industry, Shenyang, China), mandipropamid (98.8% a.i., Sigma-Aldrich Shanghai Trading Co., Ltd., Shanghai, China), azoxystrobin (95% a.i., Syngenta Biotechnology Co., Ltd., Shanghai, China), chlorothalonil (98% a.i., HeNan ChunGuang Agrochemical Co., Ltd., HeNan, China), cyazofamid (96% a.i., Sigma-Aldrich Shanghai Trading Co., Ltd., Shanghai, China), cymoxanil (98% a.i., Xinyi Agrochemicals Company, Jiangsu, China), etridiazole (92% a.i., Wanquan Agrochemicals Company, Hebei, China), fluazinam (98.4% a.i., Sigma-Aldrich Shanghai Trading Co., Ltd., Shanghai, China ), metalaxyl (96% a.i., Agrolex P. Ltd., Beijing, China), and zoxamide (97.5% a.i., Gowan Company, LLC, Yuma, Arizona, USA). These fungicides were dissolved in dimethyl sulfoxide (DMSO) to make stock solutions (10mg/ml), and the stock solutions were stored at 4°C in the dark.

Potato dextrose agar (PDA) was prepared by boiling 200 g of potato tubers for 0.5 h and filtering the material through four layers of cheese cloth The filtrate was combined with 18 g of glucose, 15 g of agar, and sufficient distilled water to increase the volume to 1 L. For carrot agar (CA), juice from 200 g of ground carrots was filtered with cheese cloth; the filtrate was combined with 14 g of agar and sufficient distilled water to increase the volume to 1 L [33].

### Isolates of *P. capsici*

During 2006–2010, stems of pepper with typical brown lesions produced by *P. capsici* were sampled from 40 locations in 24 provinces of China; as noted earlier, these locations had never been treated with pyrimorph. Small pieces of tissue (5×3 mm) cut from margins of lesions on the stem were surface disinfested in 1% NaClO (vol:vol) for 3 minutes, rinsed three times in sterile water, and placed on PDA plates amended with 50 µg/ml of rifampicin (98% a.i., Tuoyingfang Biotech Co., Ltd., Beijing, China), 50 µg/ml of penicillin (98% a.i., Tuoyingfang Biotech Co., Ltd., Beijing, China), and 50 µg/ml of pentachloronitrobenzene (40% a.i., Sanli Chemical Industry Co., Ltd., Shanxi, China). After 3 days at 25°C in the dark, mycelial plugs were cut from the colony margins and transferred to new PDA plates. A total of 226 isolates were obtained.

All the isolates were preserved on PDA slants covered with sterile mineral oil and were stored at room temperature. Single-tip transfers were made every 6 months.

### Baseline Sensitivity of *P. capsici* to Pyrimorph

The 226 *P. capsici* isolates were grown on PDA plates for 4 days at 25°C in the dark. A mycelial plug was taken from the colony margin with a cork borer (5 mm) and placed mycelia-side down in the centre of a PDA plate amended with 0, 0.1875, 0.375, 0.75, 1.5, 3, or 4 µg/ml of pyrimorph. The final concentration of DMSO was 0.1% in the medium. Each combination of isolate and concentration was represented by three replicate plates, and the experiments were performed three times. After 5 days at 25°C, the radial growth (the plug diameter was subtracted) on each plate was measured perpendicularly and averaged to calculate the percentage of growth inhibition (relative to the growth on plates without pyrimorph). EC_50_ (the effective concentration for 50% inhibition of mycelial growth) was calculated by the regression of the probit of the percentage of inhibition of radial growth against the logarithmic value of pyrimorph concentration. In addition, the variance was analyzed to determine whether the baseline sensitivity to pyrimorph differed among the locations.

### Generation of *P. capsici* Mutants Resistant to Pyrimorph

#### Mass selection from mycelial agar plugs

To obtain pyrimorph-resistant mutants from mycelial agar plugs, 10 wild-type isolates were used: Dz21, Hd3, Hd11, Hx18, Hb1-17, Mq12, N10, Pc112, Tj3-11, and Xs2. First, mycelial agar plugs (5 mm in diameter) were excised from 4-day-old colonies on PDA plates and placed mycelia-side down on PDA plates containing 20 µg/ml of pyrimorph, which was tentatively considered to be a discriminatory concentration for identifying mutants of *P. capsici* insensitive to pyrimorph. After 15–20 days at 25°C in the dark, the survivors were subsequently placed on PDA plates amended with the same pyrimorph concentration. This step was repeated until there was no significant difference in the linear growth of fast-growing sectors on the PDA plates with or without 20 µg/ml of pyrimorph. To obtain mutants with high resistance to pyrimorph, the low-resistant survivors were subjected to PDA plates amended with 100 µg/ml of pyrimorph. After 30 days at 25°C in the dark, fast-growing sectors were transferred to new PDA plates amended with the same pyrimorph concentration. This step was repeated until there was no significant difference in the linear growth of fast-growing sectors on the PDA plates with or without 100 µg/ml of pyrimorph.

For putative mutants that were resistant to 100 µg/ml of pyrimorph (as described in the previous paragraph), single-zoospore isolates were obtained using the method reported previously [34], [35]. Isolates of *P. capsici* were cultured on CA plates (9 cm diameter) for 4 days in the dark at 25°C. Then the plates were placed under florescent light with 12-h light/12-h dark at 25°C. After 5 to 7 days, when sufficient numbers of sporangia had been produced, plates were flooded with 10 ml of sterile-distilled water and placed at 4°C for 30 min and then at 25°C for 30 min. Zoospore suspensions of each isolate were spread onto 1.5% (wt/vol) water agar. After 16 h at 25°C in the dark, single germinated zoospores were transferred to PDA plates. These mutants were tested for their level and stability of resistance to pyrimorph as described later in the Methods.

#### Mass selection from zoospores

Wild-type isolates of *P. capsici* (Dz21, Hx18, Tj3-11, and Xs2) were used to select pyrimorph-resistant mutants from zoospores. For each isolate, zoospore suspensions were obtained following the method described in the previous section. The concentration of zoospore suspensions was adjusted to 10^7^/ml with the aid of a hemocytometer. The zoospore suspensions were inoculated onto PDA plates amended with pyrimorph at 1 µg/ml, at which concentration cystospore germination was completely inhibited for sensitive *P. capsici* isolates. After 7 days at 25°C in the dark, the survivors were transferred to new PDA plates with the same concentration of pyrimorph. This was repeated, and single-zoospore mutants were then generated and tested for their level and stability of resistance to pyrimorph as described in the next section.

### Characterization of Pyrimorph-resistant Mutants of *P. capsici*

#### Level and stability of pyrimorph resistance

For determination of the level of pyrimorph resistance of the mutants, mycelial plugs excised from the margins of 4-day-old colonies on PDA plates were placed upside-down on the centre of PDA plates (one plug per plate) amended with 0, 3.125, 6.25, 12.5, 25, 50, 100, or 150 µg/ml of pyrimorph. The EC_50_ values were calculated. Resistance factors (RFs) were also calculated using the following formula: RF = EC_50_X/EC_50_P, where EC_50_X is the EC_50_ value of the resistant mutant being examined, and EC_50_P is the EC_50_ value of the parental wild-type isolate of that mutant.

For assessing the stability of resistant phenotypes, the resistant mutants were subjected to 10 successive transfers on fungicide-free PDA plates. RFs were determined after the first and the tenth subculture. A “factor of sensitivity change” (FSC) was calculated as the RF value in the tenth transfer divided by that in the first transfer.

For further assessment of resistance stability, four resistant mutants were randomly selected, and 10 successive transfers with zoospores were conducted. Then, at least 50 single-zoospore isolates for each of the four resistant mutants were randomly sampled and their growth was assessed on PDA plates amended with 20 µg/ml of pyrimorph.

#### Mycelial growth as affected by temperature *in vitro*

The pyrimorph-resistant mutants and corresponding parental isolates were incubated on PDA plates at 15, 20, 25, 30, and 37°C. Colony diameters were measured perpendicularly after 5 days in the dark. Each combination of isolate and temperature was represented by four replicate plates.

#### Sporulation and cystospore germination *in vitro*

*In vitro* sporangial production and zoospore release by pyrimorph-resistant mutants and corresponding parental isolates were measured as described earlier. Zoospore production was quantified with a hemocytometer and the number of zoospores per cm^2^ of each CA plate (9 cm diameter) was calculated. After 12 h in the dark, more than 100 cystospores were examined, and cystospore germination was determined and expressed as a percentage; a cystospore was considered to have germinated if the length of its germ tube was greater than the diameter of the cystospore. These experiments were conducted at least two times.

#### Virulence and sporulation on detached bell pepper leaves

For determination of virulence of the pyrimorph-resistant mutants and corresponding parental isolates, fully expanded bell pepper leaves of the same age and area were excised from pepper plants (cultivar Tedaqiemen) with four true leaves, rinsed three times with sterile-distilled water, and placed on 1% water agar in 20-cm-diameter Petri dishes. For each isolate, 12 replicate leaves were used. A mycelial plug (5 mm in diameter) from the margin of a 4-day-old colony on PDA plates was placed on the middle of the leaf. After 4 days in a growth chamber (Shanghai Yiheng Technical Co., Ltd., Shanghai, China) with 80% relative humidity, 25°C, and 12-h light/12-h dark, the percentage of leaf areas covered by lesions was determined.

For determination of sporangia production, the 12 diseased leaves per isolate were then placed in a 50-ml centrifuge tube containing 15 ml of distilled water and mixed with a vortex apparatus (Kylin-bell Lab Instruments Co. Ltd., Haimen, China) for 15 s. The sporangia were counted using a hemocytometer, and the number of sporangia per cm^2^ of diseased leaf surface was calculated.

#### Virulence on pepper plants

Pepper seedlings (cultivar Tedaqiemen) were grown in plastic trays (540 mm×280 mm, 50 seedlings per tray) in the greenhouse. When the seedlings were 6 weeks old, they were inoculated with the pyrimorph-resistant mutants and corresponding parental strains following the method described by Sun [11], with slight modification. Zoospore suspensions of the isolates were prepared as described earlier. The seedlings in the trays were watered and then inoculated by adding 3 ml of a zoospore suspension (1×10^4^ zoospores/ml) to the soil surface around each plant. Each isolate was represented by ten replicate plants. After inoculation, the trays were placed in a greenhouse (27±2°C, 80% relative humidity, and 12-h light/12-h dark) and were watered twice daily [36]. After 7 days, virulence was assessed based on stem and leaf symptoms. Disease severity was rated on a scale of 0–5∶0, no visible disease symptoms; 1, leaves slightly wilted with black lesions beginning to appear on stems or 10–29% of the entire plant diseased; 2, 30–49% of the entire plant diseased; 3, 50–69% of the entire plant diseased; 4, 70–90% of the entire plant diseased; 5, dead plant [37], [38]. The experiment was conducted four times.

#### Control of pyrimorph-resistant mutants and wild-type isolates on bell pepper leaves treated with pyrimorph

Fully expanded bell pepper leaves of the same age were excised from glasshouse-grown pepper plants (cultivar Tedaqiemen). The leaves were rinsed three times in sterile-distilled water and placed on 1% water agar in 20-cm-diameter Petri dishes before they were sprayed with pyrimorph. For treatment of leaves with pyrimorph, pyrimorph was diluted with sterile-distilled water containing 0.1% DMSO and 0.005% Tween 20 to obtain 750 µg of pyrimorph/ml. A 1-ml volume of the pyrimorph solution (equivalent to the maximum dose of 450 g/ha in the field) or water containing 0.1% DMSO and 0.005% Tween 20 as a control was sprayed on the leaves in each dish with a hand sprayer (with 1.2×10^5^ pascals pressure). The leaves were then inoculated with wild-type isolates (Hd3, Hd11, and Dz21) or pyrimorph-resistant mutants (R3-1, R3-2, R3-3, R11-1, R11-2, and R-21) by placing one mycelial plug (obtained from the margin of a 4-day-old colony plates) on the middle of each leaf. Each isolate was represented by 12 replicate leaves. The Petri dishes with leaves were placed in a growth chamber (80% relative humidity, 25°C, 12-h light/12-h dark). After 4 days, the lesion area on each leaf was measured. The percentage of protection was calculated as % protection = 100 (1– x/y), where x and y are lesion areas on pyrimorph-treated and control bell pepper leaves, respectively.

#### Control of pyrimorph-resistant mutants and wild-type isolates on pepper plants treated with pyrimorph

Pepper plantss (cultivar Tedaqiemen) with four true leaves growing in plastic trays (540 mm×280 mm, 50 plants per tray) were treatd with 450 g ha^−1^ pyrimorph or water containing 0.1% DMSO and 0.005% Tween 20 as a control 24 h before they were inoculated with zoospore of wild-type isolates (Hd3, Hd11, and Dz21) or pyrimorph-resistant mutants (R3-1, R3-2, R3-3, R11-1, R11-2, and R-21). Suspensions of the isolates containing at least 10^4^ zoospores/ml were prepared as described earlier. A pipette was used to deliver 3 ml of each zoospore suspension to the soil surface near the roots of each plant. Each isolate was represented by ten replicate plants. The trays were placed in a greenhouse (27±2°C, 80% relative humidity, and 12-h light/12-h dark) and were watered twice daily. The severity of disease was assessed 7 days after inoculation based on a scale of 0–5 and the percentage of protection was calculated as described earlier. The experiment was performed twice.

#### Cross-resistance

The sensitivity of pyrimorph-resistant mutants and pyrimorph-sensitive isolates to fungicides with different modes of action was determined. The experiment included three CAA fungicides (flumorph, dimethomorph, and mandipropamid), azoxystrobin (a quinone outside inhibitor), chlorothalonil (chloronitrile), cyazofamid (a quinone inside inhibitor), cymoxanil (cyanoacetamideoxime), etridiazole (thiadiazole), fluazinam (an uncoupler of oxidative phosphorylation), metalaxyl (phenylamide), and zoxamide (benzamide). EC_50_ values were calculated as described earlier but with the fungicide concentrations as indicated in Table 1. Each combination of isolate and concentration was represented by three replicate plates, and the experiments were performed twice.

**Table 1:** Concentrations used to determine the sensitivity of wild-type isolates and pyrimorph-resistant mutants of *Phytophthora capsici* to various fungicides.

	Concentrations (µg/ml)	
Fungicide	For pyrimorph-sensitive isolates	For pyrimorph-resistant isolates
Pyrimorph	0, 0.1875, 0.375, 0.75, 1.5, 3, 4	0, 3.125, 6.25, 12.5, 25, 50, 100, 150
Dimethomorph	0, 0.1, 0.2, 0.4, 0.6, 0.8, 1.0	0, 0.1, 0.2, 0.4, 0.6, 0.8, 1.0, or^a^ 0, 5, 10, 20, 50, 100, 200, 400
Flumorph	0, 0.5, 0.8, 1, 1.5, 2, 3	0, 0.5, 0.8, 1, 1.5, 2, 3, or^a^ 0, 100, 200, 300, 400, 600, 800
Mandipropamid	0, 0.015, 0.02, 0.025, 0.03, 0.035	0, 0.015, 0.02, 0.025, 0.03, 0.035, or^a^ 0, 2.5, 5, 10, 20, 50, 100
Azoxystrobin	0, 0.1, 0.5, 1, 2.5, 5, 10, 15	0, 0.1, 0.5, 1, 2.5, 5, 10, 15
Chlorothalonil	0, 1, 1.5, 3, 6, 12, 25, 50	0, 1, 1.5, 3, 6, 12, 25, 50
Cyazofamid	0, 0.075, 0.15, 0.3, 0.6, 1.2, 2.5, 5	0, 0.075, 0.15, 0.3, 0.6, 1.2, 2.5, 5
Cymoxanil	0, 5, 10, 20, 40, 80, 160	0, 5, 10, 20, 40, 80, 160
Etridiazole	0, 0.5, 1, 1.5, 2, 5	0, 0.5, 1, 1.5, 2, 5
Fluazinam	0. 0.625, 1.25, 2.5, 5, 10	0. 0.625, 1.25, 2.5, 5, 10
Metalaxyl	0, 0.0625, 0.125, 0.25, 0.5, 1, 2,5	0, 0.0625, 0.125, 0.25, 0.5, 1, 2,5
Zoxamide	0, 0.02, 0.04, 0.1, 0.4, 1	0, 0.02, 0.04, 0.1, 0.4, 1

a Concentrations used to determine the sensitivity of *Phytophthora capsici* mutants with high pyrimorph resistance to dimethomorph, flumorph, and mandipropamid respectively.

### Statistical Analysis

Data were analyzed by the general linear model (GLM) procedure with Statistical Analysis System software (version 9; SAS Inc., Cary, NC). Fisher’s least significant difference (LSD) multiple range test was used to separate means. For assessment of cross-resistance, EC_50_ values were transformed to logarithmic values (logEC_50_) and subjected to Spearman’s rank correlation analysis.

### Cloning and Analysis of the *PcCesA3* Gene of the Parental Wild-type Isolates and Pyrimorph-resistant Mutants in *P. capsici*

#### Bioinformatics

For the identification of the *PcCesA3* gene in *P. capsici*, *CesA3*-specific consensus primers were designed on the basis of the multiple aligned *CesA3* sequences (the GENBANK/EMBL/uniprot data libraries) in *Phytophthora infestans* (ABP96904), *Plasmopara viticola* (ADD84672), and the genome data of *P. capsici* v1.0 (http://genome.jgi-psf.org/PhycaF7/PhycaF7.home.html). The multiple alignment was generated using ClustalW [39].

#### Cloning and analysis of the *PcCesA3* gene of the parental wild-type isolates and pyrimorph-resistant mutants in *P. capsici*

DNA was extracted from frozen mycelium by a CTAB (hexadecyltrimethylammonium bromide) procedure [40]. The *PcCesA3*-specific consensus primers were: *PcCesA3*f sequence (5′-3′) TTACTTACCGTACTCCGATCGCACG, *PcCesA3*r sequence (5′-3′) GCATGAACTCCAAACACAACAACAGC. PCRs for *PcCesA3* gene fishing experiments were performed with 50 ng of genomic DNA in a total volume of 50 µL containing 50 ng of template DNA, 1 ml of each primer (10 mM), 4 µl of dNTP mixture (2.5 mM each dNTP), 1×Easy Taq DNA Polymerase Buffer, and 2.5 U of EasyTaq DNA Polymerase (TransGen Biotech, Beijing, China).The PCR was carried out in a MyCycler™ Thermal Cycler (Bio-Rad) with the following parameters: an initial preheating for 5 min at 95°C; followed by 35 cycles of denaturation at 94°C for 30 s, annealing at 62°C for 30 s, and extension at 72°C for 4 min; and a final extension at 72°C for 10 min.

All PCR products were analyzed on 1% agarose gels, and fragments of expected size were purified from the gels with the Easy Pure Quick Gel Extraction Kit (TransGen Biotech Co., Beijing, China). The purified fragments were cloned into a pEASY-T3 cloning vector (TransGen Biotech Co., Beijing, China) and further processed following the manufacturer’s instructions. Sequencing reactions were done by Invitrogen life Technologies (Beijing, China). The PCR products were also directly sequenced for heterozygous alleles. A BLASTN search was carried out in the NCBI database (http://www.ncbi.nlm.nih.gov/) to identify correct clones. Prediction of complete *PcCesA3* open reading frames (ORFs) was done using the NCBI ORF finder program (http://www.ncbi.nlm.nih.gov/projects/gorf/). The programs in the DNAMAN software (5.2.9 Demo version) were used to predict the PcCesA3 amino acid sequences and to compare amino acid sequences of the wild-type isolates with those of the pyrimorph-resistant mutants.

### An Allele-specific PCR Method for Rapid Detection of the Novel Point Mutation in the *PcCesA3* Gene that Confers High Resistance to Pyrimorph

According to the single point mutation at codon position 1077 in the *PcCesA3* gene from pyrimorph-resistant isolates of *P. capsici*, we designed an allele-specific pair of primers. Based on the single point mutation from C to A at the codon position 1077, a nucleotide A at the 3′-end of the forward primer R1077A (5′-TCTTCGGGTTATTCGTGATGAGCA-3′) was designed to match the A at this codon position. To improve the specificity of traditional allele-specific PCR amplification [41]–[44], an artificial mismatch base T was introduced at the second nucleotide at the 3′-end of the primer, and the new primer was named R1077B (5′-TCTTCGGGTTATTCGTGATGAGTA-3′). Reverse primer R1077 (5′-CTTCGTAGTAGACCTGCCACA-3′) and non-specific forward primer CF1077 (5′-GTTCTTCGGGTTCTTCGTAATGAG-3′) were designed on the basis of the *PcCesA3* sequence.

The specificity of the allele-specific PCR primers was determined with the pyrimorph-resistant mutants (R3-2, R3-3, R11-1) and the corresponding parental isolates (Hd3, Hd11). The PCR was performed as described earlier. PCR amplification parameters were as follows: an initial preheating for 5 min at 95°C; followed by 35 cycles of denaturation at 94°C for 30 s, annealing at 58°C for 30 s, and extension at 72°C for 1 min; and a final extension at 72°C for 10 min. When the PCR was completed, a 5-µL aliquot of PCR product from each sample was analyzed by electrophoresis using a 1.5% agarose gel in TAE buffer.

## Results

### Baseline Sensitivity of *P. capsici* to Pyrimorph

**Figure 1:**
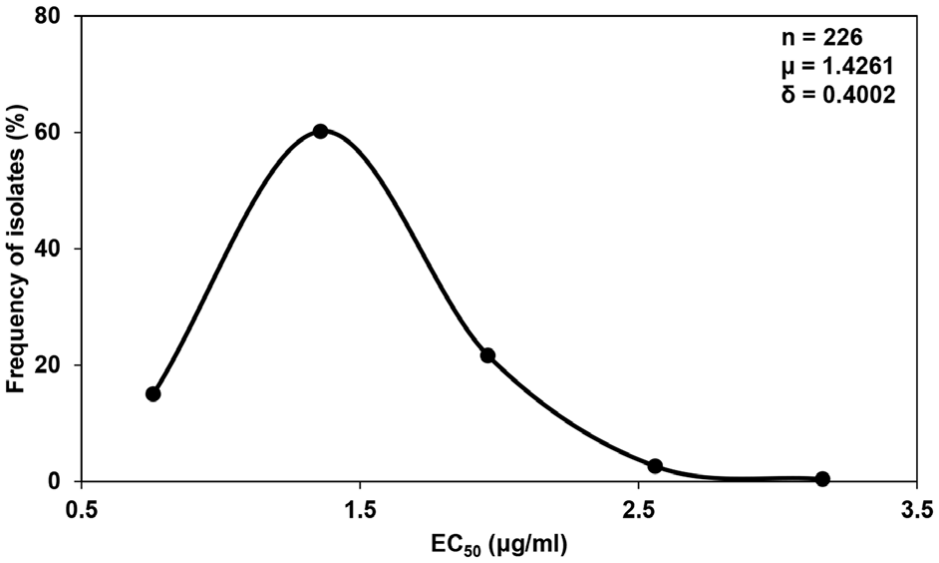
Frequency distribution of EC_50_ values of *Phytophthora capsici* for pyrimorph. A total of 226 isolates of *P. capsici* were collected from 40 locations in 24 provinces of China; the locations had never been treated with pyrimorph. EC_50_ represents the effective concentration causing 50% inhibition of mycelial growth of *P. capsici*.

A total of 226 isolates of *P. capsici* from 40 locations in 24 provinces in China were investigated (Table 2). The mean EC_50_ values varied among locations and were highest (2.0095 µg/ml) for isolates from Wuhu, Anhui Province, and lowest (1.0232 µg/ml) for isolates from Bayannuoer, Inner Mongolia Autonomous Region. However, the variation in EC_50_ values did not follow a geographical pattern (Table 2). The frequency distribution of EC_50_ values was unimodal (Figure 1), ranging from 0.7578 to 3.1610 µg/ml, with a mean of 1.4261 (±0.4002) µg/ml. The low EC_50_ values and their narrow range indicated the absence of pyrimorph-resistance among the field populations.

**Table 2:** Origin and sensitivity to pyrimorph of *Phytophthora capsici* isolates from 40 locations in China.

					EC_50_ e (µg/ml)	
Location^a^	Coordinates	Year^b^	Number^c^	Code^d^	Range	Mean
Huainan, Anhui	E117°0′16″N32°36′58″	2006	5	Hn-	1.0891–1.2325	1.1635 h–l
Wuhu, Anhui	E118°23′38″N31°19′48″	2006	7	Wh-	1.5290–2.5424	2.0095 a
Pinggu, Beijing	E117°6′32″N40°8′49″	2007	4	Pg-	0.8925–2.1328	1.6398 a–f
Shunyi, Beijing	E116°40′39″ N40°7′35″	2007	5	Sy-	0.7630–1.8208	1.3626 b-l
Dingxing, Hebei	E115°50′24″ N39°14′42″	2007	12	Hd-, Hx-	0.5725–1.6881	1.1530 h–l
Dingzhou, Hebei	E114°58′37″ N38°30′2″	2007	6	Dz-	0.9068–1.7548	1.1781 g–l
Bayannuoer, Inner Mongolia	E107°23′12″N40°45′58″	2007	6	N-	0.6943–1.3300	1.0232 l
Yangling, Shaanxi	E108°42′33″ N34°19′46″	2007	5	Sx-	1.3296–1.7485	1.5720 b–h
Fuzhou, Fujian	E119°18′32″ N26°5′29″	2007, 2010	9	Fz-, Jo-	0.9098–2.2714	1.5723 b–h
Wuhan, Hubei	E114°18′19″ N30°35′35″	2009	8	Hb-	1.0464–2.5409	1.7002 a–d
Taian, Shandong	E117°3′53″ N36°12′47″	2009	4	Ta-	1.1511–2.1991	1.6768 a–e
Taiwan	E120°56′15″ N23°43′30″	2009	3	Pc-	1.3686–2.1555	1.7723 ab
Tianjin	E117°11′48″N39°5′13″	2009	7	Tj-	0.9532–1.6836	1.2660 e–l
Kunming, Yunnan	E102°43′20″ N25°2′14″	2009	6	Xs-	0.8209–2.0889	1.6334 a–f
Minqin, Gansu	E103°4′5″N38°35′59″	2009, 2010	10	Mq-	1.1488–3.4608	1.7085 a–d
Yongzhou, Hunan	E111°36′36″N26°24′6″	2009, 2010	7	Yz-	0.9896–1.5734	1.3174 d–l
Tianshui, Gansu	E105°41′56″N34°39′36″	2010	4	Ts-	1.1905–2.4538	1.7499 abc
Zhangye, Gansu	E103°50′4″N36°3′41″	2010	5	Zy-	1.3196–1.4213	1.3611 b–l
Hengyang, Hunan	E112°38′54″N26°52′58″	2010	7	Hy-	1.2023–2.2262	1.6099 a–g
Shuangfeng, Hunan	E112°10′33″N27°26′24″	2010	4	Sf-	0.9336–1.8516	1.2768 d–l
Yueyang, Hunan	E113°7′34″N29°22′4″	2010	5	Yy-	1.4387–2.2609	1.7563 ab
Guilin, Guangxi	E110°17′30″ N25°14′42″	2010	6	Gl-	1.0362–1.6098	1.2115 f–l
Liuzhou, Guangxi	E109°23′27″ N24°25′1″	2010	4	Lz-	1.2828–1.7763	1.5581 b–i
Jiamusi, Heilongjiang	E130°18′32″N46°46′37″	2010	10	Hl-,Ym-	0.7162–1.4841	1.1254 jkl
Jiaozhou, Shandong	E120°14′18″ N36°7′34″	2010	5	Jz-	1.1628–1.5420	1.3191 c–l
Liaocheng, Shandong	E115°59′17″ N36°25′48″	2010	5	Lc-	0.9093–1.8012	1.3450 b–l
Weifang, Shandong	E119°12′45″ N36°41′42″	2010	5	Wf-	1.0766–1.2620	1.1631 h–l
Allar, Xinjiang	E81°16′48″ N40°32′46″	2010	3	Xj-	0.9455–1.1086	1.0353 kl
Tengchong, Yunnan	E98°29′53″ N25°0′40″	2010	5	Tc-	1.3138–1.8612	1.5617 b–i
Jiaozuo, Henan	E113°14′49″N35°13′41″	2010	5	Jz-	0.4575–1.7691	1.1991 g–l
Zhoukou, Henan	E114°39′11″N33°37′59″	2010	5	Zk-	1.1663–1.5745	1.3952 b–l
Nanjing, Jiangsu	E118°47′42″N32°4′16″	2010	5	Nj-	0.8103–1.4272	1.1384 i–l
Yancheng, Jiangsu	E120°9′56″N33°21′11″	2010	6	Yc-	1.3075–1.9833	1.6337 a–f
Deyang, Sichuan	E104°23′51″N31°26′49″	2010	5	Dy-	1.2823–1.4630	1.3683 b–l
Mianyang, Sichuan	E104°40′19″N31°7′59″	2010	5	My-	1.4137–1.9677	1.6815 a–e
Taizhou, Zhejiang	E121°35′28″N28°38′49″	2010	5	Tz-	1.2729–1.7017	1.4643 b–k
Changchun, Jilin	E125°19′44″N43°50′4″	2010	6	Cc-	1.0142–2.0702	1.3495 b–l
Gaoan,Jiangxi	E115°22′2″N28°23′38″	2010	3	Ga-	1.4562–1.6133	1.5563 b–j
Guiyang, Guizhou	E106°38′10″N26°39′54″	2010	5	Gz-	0.8708–1.5369	1.2126 f–l
Haikou, Hainan	E110°22′17″N19°59′56″	2010	4	Hk-	1.1113–1.7508	1.4376 b–l

a Province and city where isolates were collected.

b Year of isolation.

c Number of isolates.

d Code of isolates.

e EC_50_ = effective concentration for 50% inhibition of mycelial growth. Values followed by the same letter within a column are not significantly different according to an LSD test (*p* = 0.05).

### Generation of *P. capsici* Mutants Resistant to Pyrimorph

When exposed to pyrimorph, a total of 12 pyrimorph-resistant mutants with spontaneous mutation were obtained from parent isolates Hd3, Hd11, Hx18, and Dz21. The survival frequency, calculated as the number of resistant mutants divided by the total number of inoculations, was approximately 1×10^−4^. However, no resistant mutants were obtained from other parent isolates Hb1-17, Mq12, N10, Pc112, Tj3-11, and Xs2. Also, in spite of many attempts, no resistant mutants were obtained from zoospores of the wild-type isolates Dz21, Hx18, Tj3-11, or Xs2.

### Characterization of *P. capsici* Mutants Resistant to Pyrimorph

#### Level and stability of pyrimorph resistance

The initial EC_50_ values for the pyrimorph-resistant mutants ranged from 10.697 to 68.677 µg/ml, and the initial RF values ranged from 10.67 to 56.02 (Table 3). After 10 transfers on fungicide-free PDA, the resistance of some pyrimorph-resistant isolates had changed somewhat, as indicated by FSC values other than 1.0 (Table 3). For example, resistance increased for mutants R18-6 but decreased for R11-2, R18-3, R18-4, and R18-5 (Table 3). The pyrimorph resistance of selected single-zoospore progeny of the mutants (R11-1, R11-2, R3-2, and R3-1) was stable in that >50 single-zoospore progeny of each mutant grew well on pyrimorph-amended PDA after 10 asexual generations. These data indicate that the pyrimorph resistance of the mutants was relatively stable.

**Table 3:** Level and stability of pyrimorph resistance for the parental wild-type isolates and the resistant mutants of *Phytophthora capsici.*

		EC_50_ b (µg/ml)		RF^c^		
Isolate	Origin^a^	1^st^	10^th^	1^st^	10^th^	FSC^d^
Hd3	Parent	1.282	1.200	–	–	–
R3-1	Mutant	28.366	20.841	22.13	17.37	0.78
R3-2	Mutant	65.350	65.250	50.98	54.37	1.07
R3-3	Mutant	67.470	65.448	52.63	54.54	1.03
Hd11	Parent	1.226	1.220	–	–	–
R11-1	Mutant	68.677	64.729	56.02	53.06	0.95
R11-2	Mutant	28.979	20.393	23.64	16.72	0.71
Hx18	Parent	1.003	1.005	–	–	–
R18-1	Mutant	10.697	10.960	10.67	10.91	1.02
R18-2	Mutant	31.369	32.099	31.28	31.94	1.02
R18-3	Mutant	45.712	27.359	45.28	27.22	0.60
R18-4	Mutant	46.599	29.265	46.46	29.12	0.63
R18-5	Mutant	41.594	30.968	41.47	30.81	0.74
R18-6	Mutant	16.518	19.856	16.47	19.75	1.20
Dz21	Parent	0.907	0.906	–	–	–
R-21	Mutant	23.191	22.095	25.57	24.39	0.95

a Parent isolates were collected from the field locations. Mutants were obtained by mass selection on pyrimorph-amended medium.

b EC_50_ = effective concentration for 50% inhibition of mycelial growth at the 1^st^ transfer and the 10^th^ transfer.

c RF = resistance factor, a ratio of EC_50_ for a fungicide-resistant mutant relative to the EC_50_ for the parental isolate.

d FSC = the ratio of RF values at the 1^st^ and 10^th^ transfers.

#### Mycelial growth as affected by temperature *in vitro*

**Figure 2:**
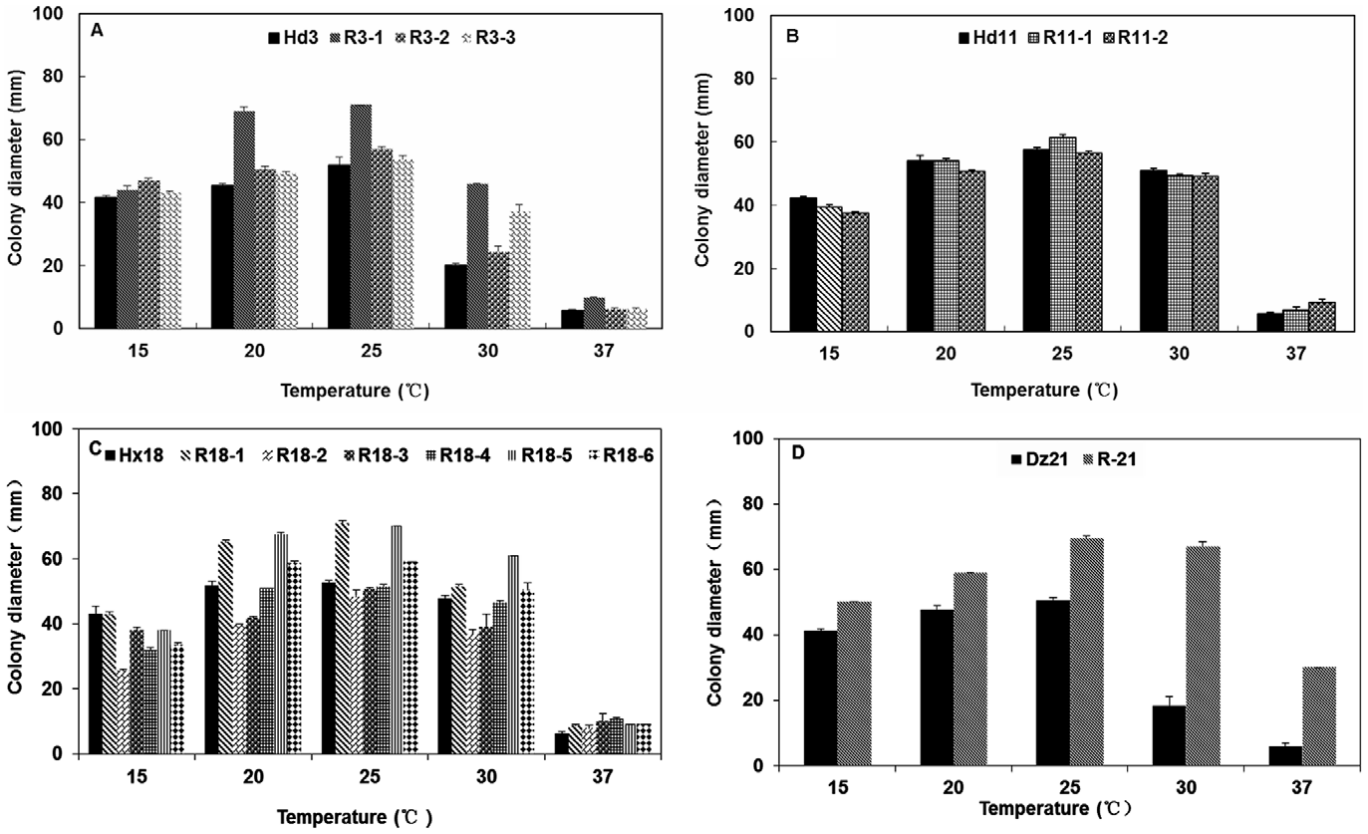
Mycelial growth of *Phytophthora capsici* isolates and mutants on PDA as affected by temperature. (A, B, C, D) Isolates designated R3-X, R11-X, R18-X, and Dz-X are mutants of Hd3, Hd11, and Hx18, respectively, and were obtained by consecutive transfers on medium amended with pyrimorph. Values are means and standard errors. Colony diameters (minus plug diameters) were measured after 4 days of growth.

For all the pyrimorph-resistant mutants and parental isolates of *P. capsici* tested, the optimal temperature for mycelial growth was 25°C (Figure 2). For Hd3 and Dz21 and their corresponding resistant mutants, the mutants grew at the same rate or faster than the parental isolates at 15, 20, 25, 30, and 37°C (Figure 2A, D); in these cases, the general pattern was that mycelial growth was greater for the mutants than for parents at all temperatures tested. For Hd11 and the corresponding resistant mutants (Figure 2B), the mutants grew at the same rate or slower than the parent isolate at 15 and 20°C but grew at the same rate or faster than their parental isolate at 25, 30, and 37°C. This indicated that the growth for this group of resistant mutants was decreased at temperatures <25°C but increased at temperatures ≥25°C. For Hx18 and its resistant mutants (Figure 2C), the mutants grew at the same rate or slower than Hx18 at 15°C but faster at 20, 25, 30, and 37°C (with the exceptions of R18-2 and R18-3 at 20 and 37°C). This indicated that mycelial growth of resistant mutants derived from Hx18 was reduced at 15°C but enhanced at temperatures >15°C. In summary, the response of the pyrimorph-resistant mutants to different temperatures varied, indicating that resistance is independent from growth characteristics at various temperatures.

#### Sporulation and cystospore germination *in vitro* and on detached bell pepper leaves

For almost all of the parental isolates and mutants that were tested, sporulation and cystospore germination for the pyrimorph-resistant mutants were lower than or similar to sporulation and germination for the corresponding parental isolates both *in vitro* and on detached bell pepper leaves (Table 4). However, cystospore germination was higher for R3-1 and R3-3 (*p*<0.05) than for the parental isolate Hd3 (Table 4).

**Table 4:** Fitness of pyrimorph-resistant mutants (mutant designations begin with the letter R) and the corresponding parental isolates of *Phytophthora capsici* a.

	*In vitro* b		*In planta* c		
Isolate	Sporulation (10^5^/cm^2^)	Cystospore germination (%)	Lesion area (cm^2^)	Sporulation (10^5^/cm^2^)	Disease score^d^
Hd3	25.0a	73.0b	5.3a	5.1b	2.5a
R3-1	1.7b	88.0a	2.4b	8.7a	2.3a
R3-2	4.2b	67.0b	3.6ab	0.6c	0.7b
R3-3	5.0b	94.0a	2.5b	0.6c	0.4b
Hd11	9.0a	90.5a	6.8a	7.0a	3.9a
R11-1	3.1b	79.3b	5.2b	3.5b	4.5a
R11-2	1.3c	88.3a	3.4c	0.5c	1.1b
Hx18	24.0a	82.0ab	9.3a	7.0a	2.8a
R18-1	4.3c	77.0b	8.7a	4.5c	0.6b
R18-2	0.8d	85.0a	4.8bc	0.7d	1.2b
R18-3	1.7d	70.5c	3.5cd	0.6d	0.1b
R18-4	0.3d	77.0b	2.6d	4.0c	0.1b
R18-5	0.4d	85.0a	5.0bc	5.1bc	0.2b
R18-6	7.0b	87.0a	5.4b	6.4ab	1.2b
Dz21	13.3a	85.0a	8.4a	6.5a	3.2a
R-21	19.5a	72.5b	6.1b	5.5a	1.7b

a Means in a column followed by the same letter are not significantly different at *p* = 0.05 according to Fisher’s least significant difference.

b On carrot agar.

c Lesion area and sporulation were determined on detached pepper leaves, and disease score was determined on growing pepper plants.

d A disease severity scale of 0–5 was used: 0, no visible symptoms; 1, leaves slightly wilted with black lesions beginning to appear on stems or 10–29% of entire plant diseased; 2, 30–49% of entire plant diseased; 3, 50–69% of entire plant diseased; 4, 70–90% of entire plant diseased; 5, dead plant [37], [38].

#### Virulence on detached bell pepper leaves and on pepper plants

Both pyrimorph-sensitive isolates and pyrimorph-resistant mutants were virulent, causing typical and severe symptoms on detached bell pepper leaves and on entire plants (Table 4). However, the size of the lesions caused by the resistant mutants was either significantly smaller than (*p*<0.05) or similar to that caused by their parent isolates on detached leaves. On entire plants, disease scores of all the resistant mutants were either less than or similar to those of the parental isolates (Table 4).

#### Control of pyrimorph-resistant mutants and wild-type isolates on bell pepper leaves treated with pyrimorph

Both the wild-type isolates and the pyrimorph-resistant mutants caused typical and severe symptoms on DMSO (0.1%)-treated bell pepper leaves (data not shown). On leaves treated with 750 µg of pyrimorph/ml, disease control was highest for the wild-type isolates, intermediate for the moderately resistance mutants, and low for the highly resistant mutants (resistance ratings were based on the EC_50_ values in Table 3). More specifically, disease control was 83, 81, and 84% for the wild-type isolates Hd3, Hd11, and Dz21; 77, 76, and 74% for the moderately resistant mutants R3-1, R11-2, and R-21; and 0, 28, and 0% for the highly resistant mutants R3-2, R3-3, and R11-1. Disease control was significantly lower (*p*<0.05) with the highly resistant mutants than with the moderately resistant mutants or the wild-type isolates.

#### Control of pyrimorph-resistant mutants and wild-type isolates on pepper plants treated with pyrimorph

For pepper plants treated with 450 g ha^−1^ pyrimorph, disease control was significantly higher (*p*<0.05) for the wild-type isolates and the moderately resistance mutants than that for the highly resistant mutants. More specifically, disease control was 72, 76, and 75% for the wild-type isolates Hd3, Hd11, and Dz21; 71, 64, and 71% for the moderately resistant mutants R3-1, R11-2, and R-21; and 7, 13, and 0% for the highly resistant mutants R3-2, R3-3, and R11-1. Pyrimorph was almost ineffective in controlling Phytophthora blight caused by the highly resistant mutants.

#### Cross-resistance

**Figure 3:**
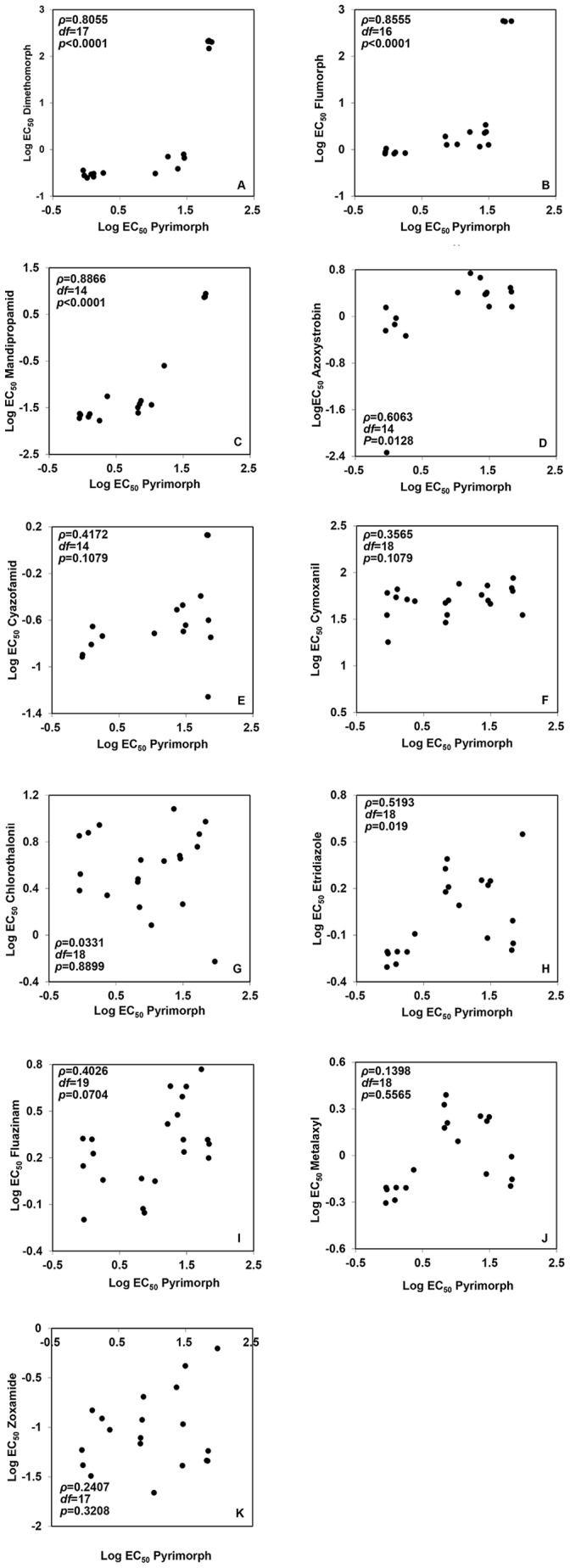
Spearman rank correlation for cross-resistance in *Phytophthora capsici* between pyrimorph and other fungicides. (A) dimethomorph; (B) flumorph; (C) mandipropamid; (D) azoxystrobin; (E) cyazofamid; (F) cymoxanil; (G) chlorothalonil; (H) etridiazole; (I) fluazinam; (J) metalaxyl; and (K) zoxamide. Data points are the logarithmic values of effective concentrations for 50% mycelial growth inhibition (log EC_50_) among *Phytophthora capsici* isolates for the indicated fungicide combinations.

EC_50_ values for pyrimorph were highly and positively correlated with EC_50_ values for the CAA fungicides dimethomorph, flumorph, and mandipropamid (Figure 3A–C); the correlation was also positive and significant for azoxystrobin (Figure 3D). Based on EC_50_ values, mutants with high resistance to pyrimorph had high resistance to the CAA fungicides dimethomorph, flumorph, and mandipropamid. However, mutants with moderate resistance to pyrimorph were sensitive to these three CAA fungicides. No cross-resistance was detected between pyrimorph and the other fungicides tested (chlorothalonil, cyazofamid, cymoxanil, etridiazole, fluazinam, metalaxyl, and zoxamide) (*p*>0.05) (Figure 3E to K).

### Cloning and Analysis of the *PcCesA3* Gene of the Parental Wild-type Isolates and Pyrimorph-resistant Mutants in *P. capsici*

**Figure 4:**
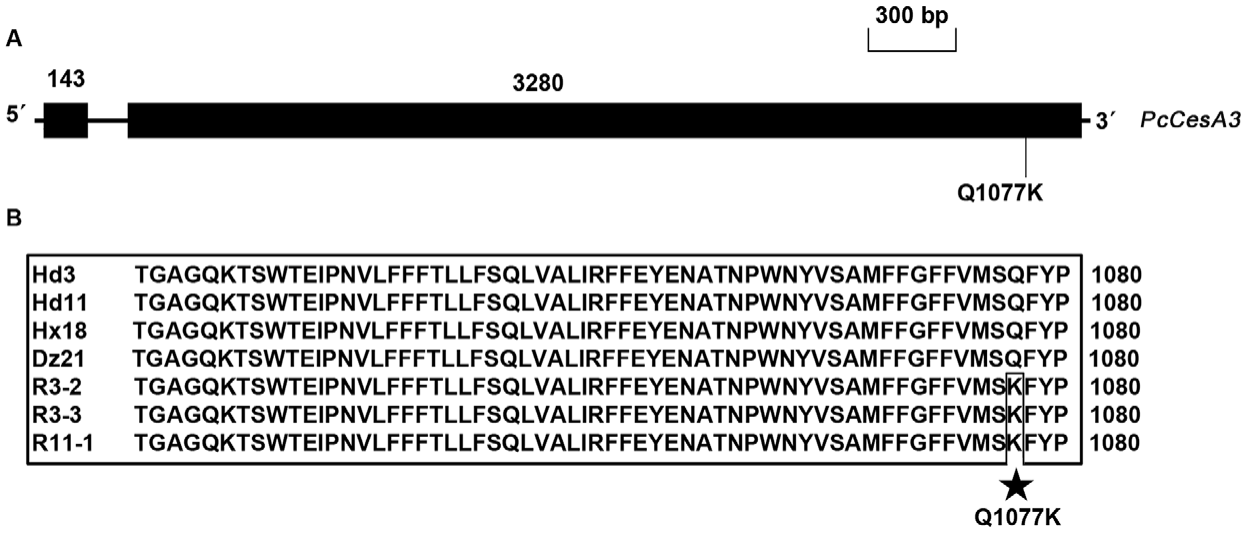
Structure and site of the mutation in the *PcCesA3* gene associated with pyrimorph resistance. (A) Intron/exon structure of the *PcCesA3* gene. Numbers represent the size in base pairs. Point mutations in pyrimorph-resistant mutants (RF>50) and the predicted amino acid substitution in the mutant gene products are indicated. (B) Alignment of partial amino acid sequences of CesA3 in *P. capsici* (PcCesA3). Hd3, Hd11, Hx18, and Dz21 are wild-type isolates. R3-2, R3-3, and R11-1 are pyrimorph-resistant mutants. Mutations in pyrimorph-resistant mutants of *P. capsici* are indicated by asterisks.

The full-length nucleotide sequences of the *PcCesA3* gene contained 3559 bp, with one 136-bp intron after nucleotide 143 (GenBank accession number JX905357, Figure 4A). The *PcCesA3* gene coded for a polypeptide chain of 1140 amino acids and had a predicted molecular weight of 126.66 kDa. A comparison of the *PcCesA3* gene in parental wild-type isolates vs. pyrimorph-resistant mutants indicated that mutation in the *PcCesA3* gene was detected only in stable, highly resistant mutants with RF values >50. The replacement of a glutamine residue (CAG) with a lysine residue (AAG) at position 1077 in CesA3 conferred stable, high resistance to pyrimorph in *P. capsici* mutants (Figure 4B).

### An Allele-specific PCR Method for Rapid Detection of the Novel Point Mutation in the *PcCesA3* Gene Allele that Confers High Resistance to Pyrimorph

**Figure 5:**
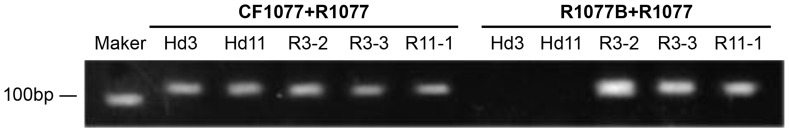
Specificity of allele-specific PCR primers for detection of *Phytophthora capsici* mutants with high pyrimorph resistance. R3-2, R3-3, and R11-1 are mutants with high resistance to pyrimorph and were obtained by exposure to the fungicide on agar; isolates Hd3 and Hd11 are the corresponding parental isolates.

The allele-specific primers, which were designed according to the single-nucleotide mutations in the *PcCesA3* gene, were used for PCR with DNA template from pyrimorph-resistant mutants and pyrimorph-sensitive isolates. PCR with the primer pair R1077B+R1077 and an annealing temperature of 58°C amplified a bright, 168-bp fragment from the pyrimorph-resistant mutants (R3-2, R3-3, and R11-1) but not from the pyrimorph-sensitive isolates (Hd3 and Hd11) (Figure 5, right). PCR with the non-specific primers CF1077 and R1077 and an annealing temperature of 58°C amplified a 170-bp fragment from both the resistant mutants and the wild-type isolates (Figure 5, left).

## Discussion

In this study, the sensitivity of 226 *P. capsici* isolates to pyrimorph was determined by measuring EC_50_ values. These isolates were collected from 40 locations in 24 provinces, covering most of the pepper-producing areas in China. Because the sampling of isolates was extensive and none of the locations had ever been treated with pyrimorph, the EC_50_ values indicate the baseline sensitivity of *P. capsici* to pyrimorph in China. In addition to having a unimodal distribution, the EC_50_ values for the 226 isolates were relatively small and had only an 4-fold range; it follows that all 226 of the field isolates can be considered sensitive to pyrimorph. Therefore, these results can be used to monitor any future development of pyrimorph resistance among *P. capsici* populations in China.

According to our data, pyrimorph resistance can be developed by culturing field isolates on agar containing pyrimorph, and the resistance appeared with a relatively high frequency of approximately 10^−4^. However, we failed to obtain pyrimorph-resistant mutants from mycelial plugs of Hb1-17, Mq12, N10, Pc112, Tj3-11, and Xs2 or from zoospores. This might be explained by genetic variation among *P. capsici* isolates. Genetic variation could be significant in this species because *P. capsici* is a heterothallic oomycete and because A1 and A2 mating types co-exist in many regions in China [45], [46]. Sexual reproduction and DNA recombination might also increase the risk of rapid development of resistance to pyrimorph in fields in China.

Other characteristics of the pathogen also affect the risk of fungicide resistance. *P. capsici* can be considered to have a low risk of resistance development based on its limited number of disease cycles per season and limited dispersal ability. Although pyrimorph-resistant isolates have not been detected in the field, all the pyrimorph resistant mutants obtained in our study can survive in the environment with some reduced fitness. We therefore suggest that the risk of resistance to pyrimorph in *P. capsici* may be low to moderate. To avoid the rapid development of resistance to pyrimorph, growers should reduce the number of pyrimorph applications and should apply pyrimorph in mixtures with multisite fungicides or with fungicides that lack cross-resistance with pyrimorph.

Only those pyrimorph-resistant mutants of *P. capsici* with RF values greater than 50 were also resistant to the CAA fungicides flumorph, dimethomorph, and mandipropamid. This suggests that if high resistance to pyrimorph develops in field populations of *P. capsici*, CAA fungicides may also fail to control the pathogen because of cross-resistance. The cross-resistance is reasonable because the resistance mechanism seems similar (but not identical) for pyrimorph and CAA fungicides. All of the mutants with a stable and high level of pyrimorph resistance in the current study displayed the Q1077K mutation in the *CesA3* gene. Previous studies reported that resistance to CAA fungicides was conferred by substitution of the following codons in the *CesA3* gene: G1105V or G1105A for *P. infestans* [28]; G1105S for *P. viticola* [30]; G1105V or G1105W for *Ps. cubensis* [29]; and V1109L for *P. melonis* [31]. The substitution of Q1077K in *PcCesA3* would therefore represent a novel mutation causing resistance to pyrimorph. The novel mutation suggests that pyrimorph and CAA fungicides might bind to somewhat different sites on the CesA3 protein. Although the changes in amino acid configuration might affect fungicide binding, the changes are evidently insufficient to prevent cross-resistance.

Unlike the *P. capsici* mutants with high pyrimorph resistance, those with moderate pyrimorph resistance were sensitive to CAA fungicides. This pattern differs from that with dimethomorph-resistant isolates in *P. capsici*, all of which are resistant to pyrimorph and to the CAA fungicide flumorph [27]. As reported here, the mutants with moderate pyrimorph resistance have no mutations in the *PcCesA3* gene. The 2,000-bp fragment upstream of the *PcCesA3* gene also does not differ between the mutants with moderate pyrimorph resistance and their parental isolates (data not shown). This indicates that pyrimorph might also bind to some unknown protein in addition to CesA3 in *P. capsici*. It is also consistent with the finding that pyrimorph has multiple modes of action including impairment of the energy generation system and impairment of cell wall biosynthesis [23].

A full understanding of the mechanism of pyrimorph resistance in *P. capsici* and the mode action of pyrimorph will require additional research. Based on this study and contrary to a previous hypothesis [27], however, pyrimorph cannot be equated with the CAA fungicides. It shares some similarities but also has important differences. In our study, also found that pyrimorph- resistant mutants were cross-resistant to azoxysrobin. The true reason has not been understood until the the biochemical mode of pyrimorph are known. At present, it might be assumed that pyrimorph also act by the inhibition of respiraton in addition to the impairment of cell wall biosynthesis in *P. capsici.*

At present, pyrimorph is an effective fungicide for the control of *P. capsici*. At the highest rate used, pyrimorph would completely inhibit the pyrimorph-sensitive and moderately resistant mutants in this study. However, even at the highest rate recommended in the field, pyrimorph would not completely control the highly resistant mutants. According to the *in vitro* and *in vivo* fitness tests reported here, and according to with our ongoing study on the competitive fitness of pyrimorph-sensitive isolates and highly resistant mutants (Hd11 and R11-1; Hd3 and R3-2) (unpublished data), mutants with high resistance can probably survive in the field although with some loss of fitness. It is therefore necessary to monitor pyrimorph resistance in *P. capsici* populations in the field. To facilitate this monitoring, we designed a molecular diagnostic method for detection of the mutation of Q1077K in pyrimorph-resistant isolates of *P. capsici*.

Compared to conventional fungicide-resistance assays, the allele-specific PCR method described here is rapid; it can determine the pyrimorph-resistance phenotype in approximately 4 h by direct sampling of diseased tissue. The AS-PCR primers designed in our study effectively identified pyrimorph resistance in *P. capsici*. Unlike the traditional AS-PCR primers, the new primers contain an additional mismatch nucleotide ‘T’ at the second nucleotide of the 3′-end; the introduction of this mismatch was previously reported to increase specificity of the allele-specific primer [31], [41]. The AS-PCR primers described here will be useful for detecting pyrimorph-resistant isolates of *P. capsici* in field populations.

As noted earlier, the pyrimorph-resistant mutants of *P. capsici* with a mutation of Q1077K were also resistant to CAA fungicides. This indicates that this newly discovered nucleotide substitution at codon position 1077 can confer CAA fungicide resistance. Although resistance based on this novel mutation has not been detected in the field, the method developed here will serve as a supplement to monitor the development of point mutations in *PcCesA3* conferring resistance to CAA fungicides in *P. capsici*.
